# Biases during DNA extraction of activated sludge samples revealed by high throughput sequencing

**DOI:** 10.1007/s00253-012-4244-4

**Published:** 2012-07-04

**Authors:** Feng Guo, Tong Zhang

**Affiliations:** Environmental Biotechnology Laboratory, Department of Civil Engineering, The University of Hong Kong, Pokfulam Road, Hong Kong, SAR China

**Keywords:** DNA extraction, Bacteria, Activated sludge, High throughput sequencing, Commercial kit

## Abstract

Standardization of DNA extraction is a fundamental issue of fidelity and comparability in investigations of environmental microbial communities. Commercial kits for soil or feces are often adopted for studies of activated sludge because of a lack of specific kits, but they have never been evaluated regarding their effectiveness and potential biases based on high throughput sequencing. In this study, seven common DNA extraction kits were evaluated, based on not only yield/purity but also sequencing results, using two activated sludge samples (two sub-samples each, i.e. ethanol-fixed and fresh, as-is). The results indicate that the bead-beating step is necessary for DNA extraction from activated sludge. The two kits without the bead-beating step yielded very low amounts of DNA, and the least abundant operational taxonomic units (OTUs), and significantly underestimated the Gram-positive *Actinobacteria*, *Nitrospirae*, *Chloroflexi*, and *Alphaproteobacteria* and overestimated *Gammaproteobacteria*, *Deltaproteobacteria*, *Bacteroidetes*, and the rare phyla whose cell walls might have been readily broken. Among the other five kits, FastDNA^@^ SPIN Kit for Soil extracted the most and the purest DNA. Although the number of total OTUs obtained using this kit was not the highest, the abundant OTUs and abundance of *Actinobacteria* demonstrated its efficiency. The three MoBio kits and one ZR kit produced fair results, but had a relatively low DNA yield and/or less *Actinobacteria*-related sequences. Moreover, the 50 % ethanol fixation increased the DNA yield, but did not change the sequenced microbial community in a significant way. Based on the present study, the FastDNA SPIN kit for Soil is recommended for DNA extraction of activated sludge samples. More importantly, the selection of the DNA extraction kit must be done carefully if the samples contain dominant lysing-resistant groups, such as *Actinobacteria* and *Nitrospirae*.

**Electronic supplementary material:**

The online version of this article (doi:10.1007/s00253-012-4244-4) contains supplementary material, which is available to authorized users.

## Introduction

Along with the development of the low cost, next generation high throughput sequencing techniques, the Earth Microbiome Project has been launched in 2011, aiming to reveal the gigantic, unexplored microbial genetic resource in soil, seawater, freshwater, the atmosphere, and other environments on our planet. At least 200,000 samples will be analyzed according to this ambitious plan. To maximize the comparability among the different studies, it needs standardized protocols for every operation step, including DNA extraction, PCR, sequencing, and data processing. Extraction of DNA of high quality is the first key step to profile microbial community with high fidelity (Martin-Laurent et al. 2001). However, the diversity of environmental sample types makes it impossible to simply apply one protocol or kit for DNA extraction.

Unlike soil or other environmental samples, activated sludge (AS) is almost composed of bacterial cells or their products, mostly extracellular polymeric substances (EPS) (Liu and Fang 2003). Generally, 1 g of dry mass of AS contains over 1–∼10 × 10^12^ bacterial cells (about 1–10 × 10^9^ cells per milliliter of working activated sludge (Nielsen and Nielsen 2001).This value is over 100-fold higher than the microbial density in soil samples. Its abundance guarantees that biomass is not a concern, and only hundreds of microliters to several milliliters of sludge are enough for DNA extraction. However, the complex biopolymers that constitute a very large portion of AS and other organic or inorganic matters adsorbed on AS are difficult to separate thoroughly from DNA during extraction. Moreover, the EPS are innate protectors of bacterial cells (Flemming et al. 2007). Breaking apart of the cell should be efficient for such samples, with the precondition that it should not result in over-fragmentation of DNA.

To the best of our knowledge, there is currently no well-accepted commercial DNA extraction kit designed for AS samples, which is distinct from all other environmental samples. Thus, the cross-use of commercial kits made for other sample types (such as soil and stool) should be evaluated for their applicability to AS samples, although they had been randomly selected in previous AS studies. On the other hand, for AS samples containing bulking water, ethanol fixation is usually adopted during transportation and storage. As far as we know, the effect of this processing on the bacterial community profiling has not yet been evaluated.

Besides its unique nature, AS also contains extremely diverse bacterial species. Over 15 phyla could be usually found within one single AS sample (Zhang et al. 2012). Thus, AS had been adopted as good material for molecular methodological assessment in FISH and terminal restriction fragment length polymorphism (Wagner et al. 1993; Liu et al. 1997). Several evaluation studies on DNA extraction kits or methods for AS samples have been performed in recent years (Vanysacker et al. 2010; Bushon et al. 2010; Bonot et al. 2010). However, these studies never tested the effectiveness of different kits by high throughput sequencing and, thus, were based on detailed taxonomic information, which is the more important index for community structure analysis. In the present study, seven commercial kits for DNA extraction were evaluated for their effectiveness on ethanol-fixed or fresh as-is (unfixed) AS samples. Besides yield and purity, the bacterial community for each extracted DNA was evaluated by deep sequencing. Unprecedented sequencing depth helps us to get detailed information of dominant and take a glimpse on subdominant and rare taxons, which maybe also playing some significant roles in the community. The results are valuable not only for judging the optimal kit for AS samples but also for evaluating the potential biases caused by different DNA extraction kits, which should be of concern when dealing with various environmental samples.

## Materials and methods

### Activated sludge samples

Two AS samples were collected from the Stanley sewage treatment plant (STP) and the Shatin STP located at Hong Kong, China. The former is a normal municipal wastewater treatment plant, while the latter treats saline wastewater (because of seawater toilet-flushing in Hong Kong) with about 1.1 % salinity. The fluorescent images for the two samples (stained by SYBR green I) are shown in Figure S1, which indicates that both the AS samples are not bulking. The sludge samples were transported to the lab within 2 h, and then portions of them were fixed at a 1:1 ratio with absolute ethanol and then stored at –20 °C for 12 h. For each sludge sample, the unfixed fresh as-is sludge was washed twice with 0.9 % NaCl solution and resuspended in an equal volume of 0.9 % NaCl solution. Then, 1.5 mL of the unfixed samples was transferred into each of 30 microcentrifuge tubes, using wide-mouthed pipette tips (allowing transfer of large particles in the samples). The tubes were centrifuged at 10,000 rpm for 5 min, and the supernatants were discarded. The ethanol-fixed samples were concentrated to half the volume (all tubes containing equal biomass for each sample) and then processed as the unfixed samples after a 12 h fixation. Finally, all pellets were stored at –80 °C until DNA extraction. The dry weight of the sludge used for DNA extraction was recorded for both samples (*n* = 4).

### DNA extraction

Seven commercial DNA extraction kits were evaluated in this study. Their names, abbreviations, and some of their characteristics are summarized in Table 1. There were five kits for soil samples, that is, MoBio PowerSoil^@^ DNA Isolation Kit (MoBio Laboratories, Inc., USA), MoBio UltraClean^@^ Soil DNA Isolation Kit (MoBio Laboratories, Inc., USA), FastDNA^@^ SPIN Kit for Soil (Qbiogene, Inc., CA), ZR^TM^ Soil Microbe DNA Kit (Zymo Research Corporation, USA), and EPICENTRE^TM^ Soil Master DNA Extraction Kit (Epicentre^@^ Biotechnologies, USA), plus two kits for stool samples, that is, MoBio UltraClean^@^ Fecal DNA Isolation Kit (MoBio Laboratories, Inc., USA) and QIAamp DNA Stool Mini Kit (Qiagen, Germany). All operations were conducted according to the instructions of each kit, except for the following: (1) If there is a bead-beat step, the Fast bead beater (FastPrep^@^-24, MP Biomedicals, USA) was adopted for all kits with the setting at 6.0 m s^−1^ and 5 × 1 min duration; (2) the volume of the elution buffer was 100 μl in the final elution. For each sample (including fixed and as-is) and each kit, three replications were performed. Thus, a total of 84 treatments were conducted.

**Table 1:** Seven DNA extraction kits evaluated in this study

Kits	Abbreviation	Cell lysis	DNA purification	References
MoBio	MB-FE	BB and CLB	Spin filter	McGarvey et al. 2004
UltraClean^@^ Fecal DNA Isolation Kit				
MoBio	MB-PS	BB and CLB	Spin filter	Zhang et al. 2009
PowerSoil^@^ DNA Isolation Kit				
MoBio	MB-US	BB and CLB	Spin filter	Gelder et al. 2005
UltraClean^@^ Soil DNA Isolation Kit				
Qbiogene	FA-SS	BB and CLB	Spin filter	Auerbach et al. 2007
FastDNA^@^ SPIN Kit for Soil			Matrix binding DNA specifically	
Qiagen	QG-ST	CLB	Spin filter	Bonot et al. 2010
QIAamp DNA Stool Mini Kit				
Epicentre^TM^	EP-SM	CLB	Spin filter	Roh et al. 2006
SoilMaster DNA extraction Kit			Adsorb inhibitors with matrix	
ZR^TM^	ZR-SM	BB and CLB	Spin filter	Wang et al. 2011
Soil Microbe DNA Kit			Adsorb inhibitors with matrix	

*BB* bead beating, *CLB* cell lysis buffer

### DNA examination

For DNA quantification, two methods were adopted, i.e., NanoDrop (NanoDrop-1000, Thermo Scientific, USA) and Qubit (Invitrogen, USA, using the high sensitive DNA quantification kit), with the detection limits of 2 and 0.1 ng μl^−1^ respectively. Two microliters of each sample for NanoDrop was loaded directly after extraction. For Qubit, the DNA was diluted 20–200 times in the working solution according to the concentration. After quantification, 8 μl DNA was loaded onto a 1.0 % agarose gel containing 1× GelRed dye and 1× TAE buffer. DNA was allowed to run for 30 min under a voltage of 100 V. The gels were visualized in the Bio-Rad Gel DOC system (Bio-Rad Laboratories, Inc., USA).

### PCR and Illumina high throughput sequencing

For Illumina high throughput sequencing, the highest yields of DNA extracted from each kit for each sample (both fixed and as-is) were evaluated. The V6 region of the 16S rRNA gene was amplified by the primer set of V6F and V6R (Sogin et al. 2006). The forward primer was added with 28 sample-specific, eight-base barcodes at its 5′ end, which allows the multiplexing during sequencing (Binladen et al. 2007). A final concentration of 0.5 ng μl^-1^ genomic DNA was used as a template because some kits produced a very low concentration of DNA. PCR conditions were set as follows: 95 °C for 5 min, then 30 cycles of 95 °C for 15 s, 57 °C for 30 s, and 72 °C for 30 s, finally extending at 72 °C for 10 min. Three 50-μl PCR reactions were conducted for each DNA sample and then mixed and visualized in a 2 % agarose gel after electrophoresis (∼100 bp). Then, the products were purified with a PCR production purification kit (PCR quick-spin^TM^ kit, iNtRON Biotechnology, Inc., Korea). Finally, the PCR products from each treatment were mixed to obtain equal molar DNA for sequencing. About 18 μg PCR products were sent out to BGI (Shenzhen, China) for 101PE paired-end sequencing on the platform of Illumina Hiseq2000 (Illumina, USA).

### Sequence processing

The raw paired-end sequence data analysis was performed as follows: (1) we combined each pair-end reads into one sequence and removed all sequences with any mismatches between the two reads (using a self-written python script); (2) we removed sequences without barcodes and obtained the tags containing barcode and primer (using a self-written python script); (3) we trimmed and cleaned the subsample and get operational taxonomic units (OTUs) from the tags through the Mothur software (see SI methods in detail) (Schloss 2009; Schloss et al. 2009); (4) we extracted one representative sequence from each OTU (using a self-written python script) and classified it through the GAST program (Huse et al. 2008).

## Results

### DNA quantification and qualification

**Fig. 1:**
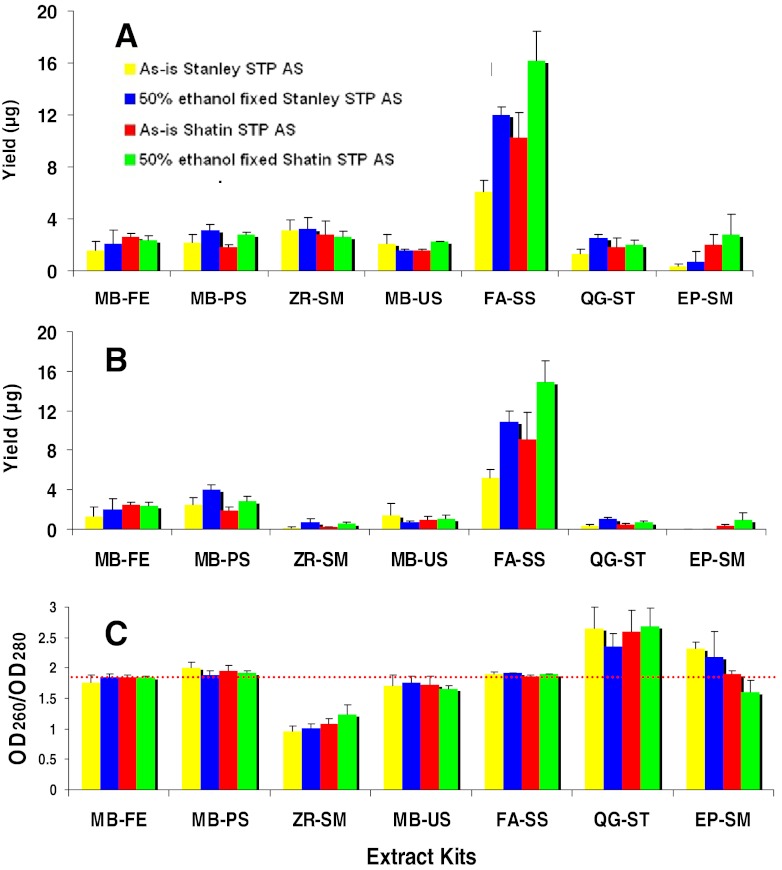
The DNA yields and purity of the two AS samples with the seven kits. **a** DNA quantified with NanoDrop. **b** DNA quantified with Qubit. **c** DNA qualified by OD_260_/OD_280_ with NanoDrop. The *dashed line* shows the ratio at 1.85, which is the index of optimal DNA purity

The yields of extracted DNA are shown in Fig. 1a and b. The dry weights of sludge used in extraction were 2.3 ± 0.4 and 3.5 ± 0.6 mg for Stanley and Shatin samples, respectively. The highest yield of DNA was obtained by the FA-SS kit, which was more than two times that of the second highest kit (MB-PS). The three MoBio kits had moderate yields (0.7–4 μg). The DNA contents extracted by the other three kits were too low to be observed in the agarose gel (Figure S2). On the other hand, samples fixed in 50 % ethanol produced significantly more DNA than the corresponding unfixed ones in FA-SS and MB-PS treatments (*P* < 0.05).

The Qubit quantification results were usually lower than those obtained using NanoDrop, especially for DNA extracts of low quality, as shown in Fig. 1c. Qubit results based on fluorescence may be more reliable because the impurities in the DNA extract could also result in UV absorbance, whereas fluorescence-based quantification was more specific. According to the results of the Qubit method, the yield of the FA-SS kit was 2,241 to 4,741 μg/g dry mass, relatively higher than that in a previous report (Bonot et al. 2010).

Two kits, i.e., QG-ST and EP-SM, without bead-beating to disrupt cells, yielded very low DNA (<0.7 μg in all treatments), showing that robust mechanical homogenization is needed for DNA extraction of AS samples.

By contrast, the FA-SS and MB-FE kits produced the purest DNA indicated by the OD_260_/OD_280_ values of ∼1.85 in all treatments. The highly purified DNA extracted by the FA-SS kit implied that the purified method of this kit was more efficient and robust. The ZR-SM was very low in purity, with the ratio of OD_260_/OD_280_ around 1.0. It was in accordance with the significant difference between the DNA amounts determined by NanoDrop and Qubit. The two MoBio kits, MB-FE and MB-PS, obtained fairly pure DNA, with slight variations. The high ratio of OD_260_/OD_280_ of the DNA extracted from QG-ST and EP-SM may result from the low DNA concentration that causes imprecision in absorption measurements. Ethanol fixation did not affect the purity for all treatments (*P* > 0.05 in all kits). The results of DNA electrophoresis are shown in Figure S2. DNA obtained after all the treatments (QG-ST, EP-SM, and ZR-SM were very weak) was smaller than 21 kb, typically around 10 kb.

### OTU-based analysis

About 3.7 million raw reads were obtained by the high throughput sequencing. After processing, all treatments were subsampled at the same depth of 46,734 tags. The two treatments of Shatin AS extracted by the EP-SM kit with and without fixation were excluded because of the low read number. There were 29,553 OTUs for a total 1,215,084 tags (a total of 26 treatment groups). The OTU numbers were 17,872 and 15,079 OTUs for the Stanley and Shatin AS samples, respectively. The rarefaction curves for the unfixed and fixed treatments in each activated sludge sample are shown in Figure S3.

Table 2 lists the analysis of the number of OTUs of each DNA extraction treatment. The treatments with the highest number of OTUs were ZR-SM and MB-PS, whereas the treatments with the lowest number of OTUs were EP-SM and FA-SS for the Stanley and Shatin AS samples, respectively. The treatments with the least OTUs usually had smaller diversity indexes calculated based on the total OTUs. However, the numbers of abundant OTUs containing over 100 tags in each treatment, as well as the diversity indexes calculated based on the top 500 OTUs, were not obviously different among the five kits with the bead-beating step. The two kits without the bead-beating step were obviously lower in the numbers of abundant OTUs and diversity indexes based on the top 500 OTUs than the other five kits. The missed OTUs within the top 500 OTUs also indicated that the EP-SM and QG-ST kits were less efficient than the other five kits, whereas the five kits had little difference between them.

**Table 2:** Total OTUs and OTU-based diversity indexes of the different extraction treatments

Sludge	Kit	Fixation	Total OTUs	>100 tags OTUs	Diversity^a^	Diversity^b^	Number of undetected Top 500 OTUs (the highest rank of the missing OTUs)^c^
Stanley	MB-FE	UF	4,359	85	6.107	5.156	2 (339)
	MB-FE	F	3,895	89	6.1	5.262	3 (291)
	MB-PS	UF	4,243	86	6.206	5.306	1 (339)
	MB-PS	F	3,884	88	6.116	5.282	1 (339)
	MB-US	UF	4,173	86	6.123	5.172	3 (280)
	MB-US	F	4,451	83	6.289	5.242	0
	FA-SS	UF	3,814	81	5.925	5.124	1 (339)
	FA-SS	F	4,140	82	6.145	5.27	2 (339)
	QG-ST	UF	3,916	70	5.811	4.84	18 (212)
	QG-ST	F	3,579	77	5.638	4.805	31 (119)
	EP-SM	UF	3,382	76	5.716	4.909	21 (119)
	EP-SM	F	3,513	75	5.763	4.924	29 (265)
	ZR-SM	UF	4,722	88	6.427	5.377	0
	ZR-SM	F	4,414	80	6.252	5.297	1 (500)
Shatin	MB-FE	UF	3,943	92	6.175	5.334	1 (290)
	MB-FE	F	3,751	92	6.069	5.276	1 (290)
	MB-PS	UF	3,996	86	6.121	5.256	1 (290)
	MB-PS	F	3,972	82	6.142	5.289	1 (290)
	MB-US	UF	3,902	88	6.13	5.285	1 (290)
	MB-US	F	3,519	92	5.906	5.125	3 (290)
	FA-SS	UF	3,429	93	5.914	5.208	2 (290)
	FA-SS	F	3,410	89	5.933	5.234	1 (290)
	QG-ST	UF	3,515	77	5.846	4.97	20 (164)
	QG-ST	F	3,639	82	5.956	5.112	15 (152)
	ZR-SM	UF	3,783	84	5.986	5.179	1 (290)
	ZR-SM	F	3,824	86	5.975	5.168	1 (480)

^a^Shannon diversity indexes calculated on the basis of total OTUs

^b^Shannon diversity indexes calculated on the basis of the top 500 OTUs

^c^Top 500 OTUs were determined based on the total abundance in all treatments for each sample

**Fig. 2:**
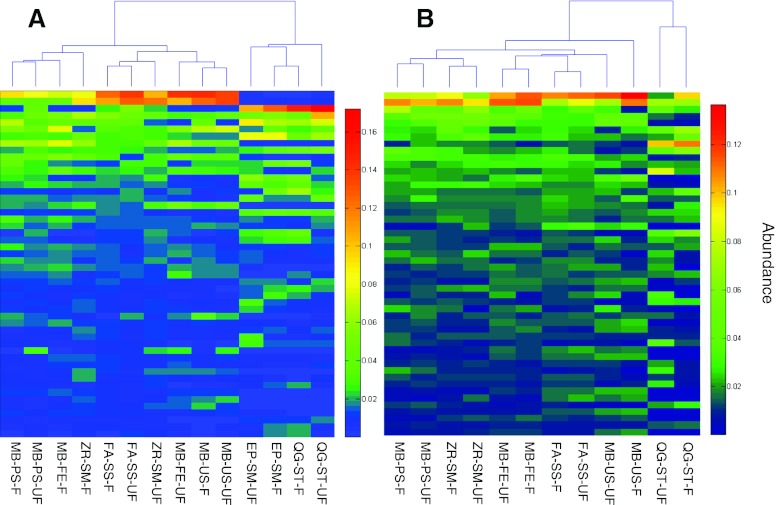
The top 50 OTU-based clustering among different treatments of the Stanley AS sample (**a**) and the Shatin AS sample (**b**). The top 50 OTUs were determined by their total abundances in all treatments for each AS sample. The treatments were clustered based on the Bray–Curtis distance, calculated by the relative abundance of each OTU to the total tags of the top 50 OTUs. Generally, the top 50 OTUs occupied about 40–50 % of the total tags. The heat maps were drawn using the MATLAB software

The cluster based on abundances of top 50 OTUs among different treatments for each sample is shown in Fig. 2. For the Stanley AS sample, the two kits, QG-ST and EP-SM, and the other five kits were clustered together, respectively. For the Shatin AS sample, the two treatments for each kit were clustered separately (except for ZR-SM), and then the five kits with the bead-beating step and the other two kits clustered together, respectively. This observation indicates that the kit was more determinative than the fixation treatment and other biases generated during PCR/sequencing. The MB-US and FA-SS kits had the minimum between their fixation and nonfixation treatments for the Stanley and Shatin AS samples, respectively. Moreover, in the OTU-based heat maps of the two samples shown in Fig. 2, the lower the number of blue blocks (indicating low abundance) in the high-ranked OTUs, the more reliable the kits. Therefore, in general, the MB-PS-UF, ZR-SM, and the FA-SS treatments were more genuine than the other kits for both samples. The QG-ST and EP-SM were obviously unreliable because of the large numbers of blue blocks in high-ranked OTUs.

### Taxonomy-based analysis

**Fig. 3:**
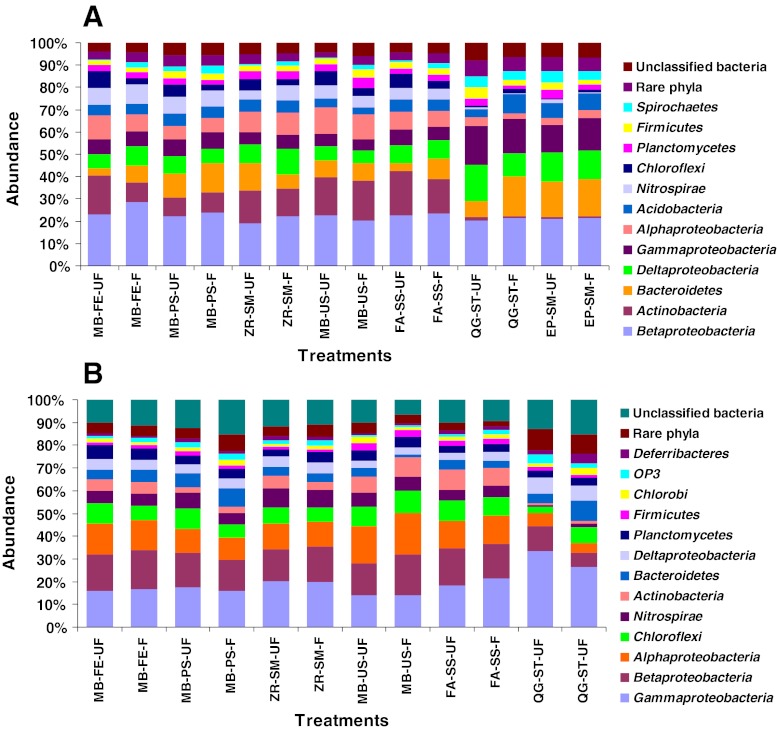
The bacterial community structures (at the phylum level) of samples from the Stanley (**a**) and Shatin (**b**) STPs using different kits. The presented phyla were dominant groups that were over 1 % in abundance

The bacterial community structure at the phylum level for each treatment is shown in Fig. 3. The most abundant phyla (here and below, the Alpha, Beta, Gamma, and Delta classes in *Proteobacteria* were treated as phyla) were *Betaproteobacteria* and *Gammaproteobacteria* for the Stanley and Shatin AS samples, respectively. The treatments of QG-ST and EP-SM without the bead-beating step resulted in a significantly low abundance in Gram-positive *Actinobacteria*, *Nitrospirae*, *Alphaproteopbacteria*, and *Chloroflexi*, especially for the former two phyla. The other Gram-positive phylum, the *Firmicutes*, was not obviously different among all the treatments. This indicated that only chemical or enzymic lysis could not disrupt efficiently the Gram-positive *Actinobacteria* and that robust mechanical homogenization is needed. However, it seemed that *Gammaproteobacteria*, *Deltaproteobacteria*, *Bacteroidetes*, and many rare phyla were usually overestimated in the QG-ST and EP-SM kits with gentle cell lysis.

Moreover, the 50 % ethanol fixation slightly changed the community structure. A detectable bias was *Chloroflexi* in the Stanley sample. Fixation decreased the abundance of this phylum. This phylum usually has a filamentous shape, and the reason for such a decrease is not clear.

**Fig. 4:**
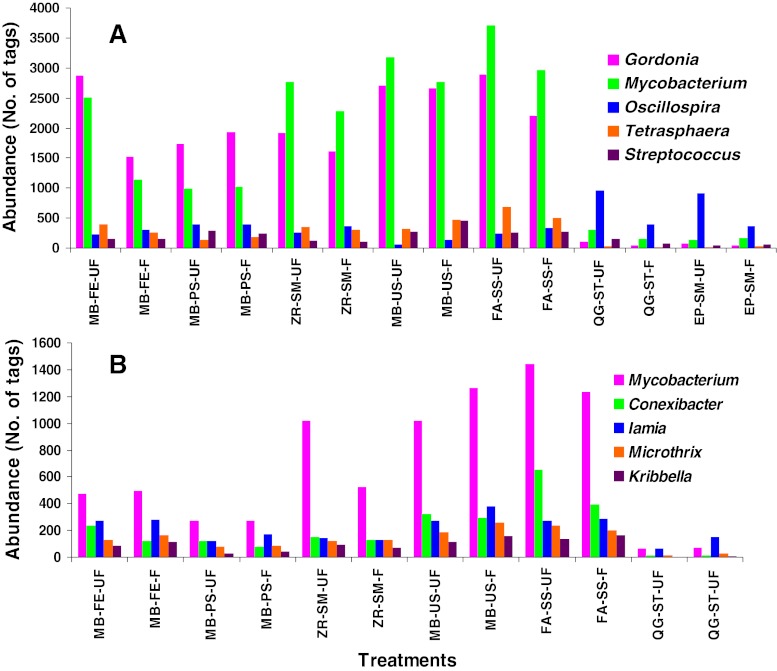
The abundances of the top 5 Gram-positive genera in different treatments in AS from the Stanley (**a**) and Shatin (**b**) STPs. Seven of the nine genera belong to high a G+C phylum, *Actinobacteria*, and the other two, *Oscilliospira* and *Streptococcus*, are *Firmicutes*

To further investigate the efficiencies of cell lysis of the various kits, the abundances of the top 5 Gram-positive genera were investigated, and the results are shown in Fig. 4. First, the treatments without the bead-eating step (i.e., QG-ST and EP-SM) had very low abundances of the top 5 Gram-positive genera in both the samples. Second, among the five kits with the bead-beating step, the FA-SS kit exhibited the best capability for cell lysis among the top 5 Gram-positive genera. The ZR-SM and MB-US kits also worked well. However, the MB-FE and MB-PS did not perform very efficiently, as indicated by the low detected abundances of these genera. Interestingly, the third abundant Gram-positive genus in the Stanley sample, *Oscillospira*, was richer in the treatment with QG-ST and EP-SM than that with the other five kits.

## Discussion

Unlike other environmental samples, activated sludge is composed of nearly all microbial cells and their products (Frølund et al. 1996). The cells cluster together and are enclosed with EPS, which can protect them from shear forces and chemical reagents including sodium dodecyl sulfate (Davies et al. 1998). In terms of productivity and diversity, results from this study showed that the mechanical homogenization (the bead-beating step) is obviously necessary for DNA extraction from sludge samples.

The factors that affect DNA yield for a kit are mainly the efficiency of the cell lysis step and the subsequent losses during purification. The five kits with the bead-beating step have minor differences in the lysis process except that the FA-SS kit contains glass beads with different sizes (0.1–1 mm in diameter). The big glass beads may be efficient for dispersing cells from clusters, and the small ones are dedicated to crush the cells. However, the FA-SS kit also contains a unique matrix that specifically binds DNA, whereas all of the other six kits just adopt a spin column to bind DNA. The two unique designs in the FA-SS kit may promote the quantity and quality of the extract DNA from AS, as indicated by the result.

The quantity of DNA is usually not of great concern for PCR-based community analysis because even as low as 10–100 ng DNA (equals to about 10^6^–10^7^ cells) is already enough for amplification and then sequencing. However, for the current metagenomic study sequenced by the Illumina platform, 3–10 μg virginal, highly pure DNA is needed. This makes the FA-SS and MB-PS the only two candidate kits. Dramatically, our result showed that the low quantity and even the low quality of the extracted DNA could also provide a fair profile of the bacterial community. For example, the ZR-SM kit produced very low concentrations of DNA with low quality (ratio of OD_260_/OD_280_ around 1.0), but the OTU-based and taxonomic analysis indicated that the results reflected reasonably the major bacterial community profile, with only a slight difference from the three MoBio kits and FA-SS kit. However, the QG-ST and EP-SM kits that also extracted low quality and quantity of DNA showed much higher biases on the community profile compared with the other five kits. This suggests that the ZR-SM kit may be efficient in cell lysis, but loses much DNA during the subsequent purification steps, which is a random event. Thus, it does not affect the community structure. It is noteworthy, however, that all the kits could be utilized efficiently by changing some of the operations. For example, the EP-SM kit performs the centrifugation at 1,000–2,000 g in some cases, which may be fair for soils (the density is much higher than activated sludge), but unsuitable for sludge samples, and could cause loss of sample. Increasing the strength of the centrifugation may increase the yield for this situation.

Other than yield and fidelity, a co-existing problem is that the DNA extract from commercial kits are usually small in segment size. This may be the result of the high shear force during the bead-beating or vortex processing. Small pieces of DNA are not suitable for construction of the fosmid, cosmid, and BAC libraries that prefer genomic DNA fragments over 25 kb, which are usually extracted by lab-developing methods (Robe et al 2003). Moreover, if the extracted DNA is used in full-length 16S rRNA gene (∼1.5 kb) amplification, ∼10 kb-sized fragments theoretically lose about 15 % of the genes. However, the current high throughput sequencing will be little affected because of the short amplified regions (mostly <400 bp).

For environmental samples, especially for those containing bulking water, fixation is needed before long-term transportation and storage. DNA may be altered in two different ways without fixation: (1) The bacterial community may change rapidly during transportation and storage because of the change of environment, and (2) DNA may leak out from cells that die during transportation and storage into the bulking water and then be washed away. For sludge samples, fixation in 50 % ethanol (final concentration) was recommended, which is the same as sample fixation for fluorescence in situ hybridization (FISH) (Xia et al. 2007). Another advantage is that 50 % ethanol would not be frozen at –20 °C. The results in this study showed that the fixation could improve DNA yield, although the reason is unclear. In addition, most of the slight variation of the bacterial community between the fixed and nonfixed treatments could not be attributed to the fixation. It could arise from the biases of PCR or sequencing.

The total OTU number and the diversity indexes based on the total OTUs could not be the key criteria for the evaluation of the efficiencies of DNA extract kits, especially under the conditions that not all species were detected by sequencing at insufficient depth, considering the extremely high diverse bacteria in activated sludge. In fact, under 46,734 sequencing depth, the ∼4,000 OTUs in each treatment usually had about 50–70 % singletons and > 90 % OTUs containing <10 tags (data not shown), which were obviously rare groups with little significance, having abundances of 0.002–0.02 %). The more OTUs and higher diversity indexes may represent more bacterial species at the price of biases on the abundances of the dominant or subdominant groups if the kits could not extract DNA effectively from certain such groups. On the contrary, the top 500 and 50 OTUs usually accounted for more than 80 and 50 % of total tags, respectively. Thus, they are more suitable to evaluate the efficiency of the kits. Under these conditions, the five kits with the bead-beating step are significantly better than the two kits that only used lysis buffer. However, the differences among the five kits need to be determined by taxonomic analysis.

The Gram-positive bacteria are resistant to both detergents and mechanical resistance because of their thick cell wall (Bollet et al. 1991) or because some of them can form spores (Kuske et al. 1998). Therefore, it could be simply considered that the more Gram-positive bacteria are detected, the more efficient the DNA extraction kits are. In terms of this, the most efficient two kits were FA-SS and MB-US because more Gram-positive *Actinobacteria* were detected in the two treatments at both the phylum and genus levels than the others. *Actinobacteria* is an ubiquitously dominant phylum in AS and plays key roles in polymer degradation, glycogen accumulation, and polyphosphate accumulation (Seviour and Nielsen 2010). A study that used untreated sludge to perform PCR and cloning could not detect *Actinobacteria*, although about 13 % of the cells belonged to this phylum, as determined by FISH (Snaidr et al. 1997). Another study that adopted denaturing gradient gel electrophoresis as the DNA extraction evaluation method treated this high G + C phylum as a key indicator for DNA extraction methods (Niemi et al. 2001). All these suggested the abundance of *Actinobacteria* could be a key sign for efficiency of DNA extraction, especially for cell lysis. Recently, a high-throughput sequencing, metagenomic study of AS found biases when comparing the sequencing data with the results from the FISH method (Albertsen et al. 2011). Very interestingly, the study also found that the *Actinobacteria* and *Chloroflexi* are seriously underestimated in the metagonomic data comparing with the FISH results, although the FA-SS kit was adopted. A flaw emerged that bead-beating was performed only for 3 × 5 s. This observation indicates that the operational time for bead-beating should also be concerned. By contrast, the detection of the other Gram-positive phylum, *Firmicutes*, was minimally affected by different kits, even for the two inefficient kits. Also interestingly, a genus that belongs to the *Firmicutes*, *Oscillospira*, was more abundant in treatments of the two inefficient kits. This observation indicates the different efficiencies of the kits in detecting various subgroups of Gram-positive bacteria. The other underestimated phylum, *Nitrospirae*, has a special wide periplasmic space, which is near twice that in other Gram-negative bacteria (Watson et al. 1986). This structure may hinder the release of DNA following inefficient cell lysis treatment. Similarly, DNA from *Chloroflexi* was hard to extract, possibly also because of the layered cell envelopes (Sutcliffe 2011). However, the reason for the underestimation of *Alphaproteobacteria* is unclear.

Moreover, the results from the inefficient kits are also valuable because they imply which groups tend to be overestimated. In this study, the *Gammaproteobacteria*, *Deltaproteobacteria*, *Bacteroidetes*, and many rare phyla were overestimated in the two kits with low efficiency. This implies that bacteria within these groups are more likely to be destroyed and, thus, overestimated if inefficient methods are adopted to disrupt all the bacterial cells. Since the usual high richness of *Gammaproteobacteria* and *Bacteroidetes* in many environmental samples was found, it is noteworthy whether they were overestimated by the inefficient DNA extraction to some extent.

In summary, in the light of our results, the FastDNA^@^ SPIN kit for Soil is recommended for DNA isolation of activated sludge samples because of its high yield, purity, and excellent cell-breaking capability. Although the number of total OTUs from this kit was not high, the major groups and Gram-positive bacteria that were identified indicated its reliability compared to other kits. The three MoBio kits and ZR-SM kit were also fair, but insufficient in yield and/or purity, which are essential for current metagenomic studies. In addition, the results also proved that the bead-beating step is necessary for activated sludge samples because some phyla, such as *Actinobacteria* and *Nitrospirae*, are significantly resistant to the simply chemical cell lysis treatment. Careful selection of extraction kits or methods should be considered if these phyla would exist dominantly in certain environmental samples.

## Electronic supplementary material

Below is the link to the electronic supplementary material.

ESM 1 (PDF 2002 kb)
