# Supplementary material for: Biases during DNA extraction of activated sludge samples revealed by high throughput sequencing
Source: Appl Microbiol Biotechnol. 2012 Jul 4;97(10):4607–16. doi: 10.1007/s00253-012-4244-4 (PMC3647099; doi:10.1007/s00253-012-4244-4)
Supplement: Supplementary file 1 — (PDF 2002 kb) [file 253_2012_4244_MOESM1_ESM.pdf]

Electronic Supplementary Materials for

Biases during DNA Extraction of Activated Sludge Samples Revealed by High Throughput Sequencing

Feng Guo and Tong Zhang*

Environmental Biotechnology Laboratory, The University of Hong Kong, Hong Kong SAR, China

Address: Environmental Biotechnology Lab, Department of Civil Engineering, The University of Hong Kong, Pokfulam Road, Hong Kong SAR, China

*Correspondence author

Email: zhangt@hku.hk

Tel: +852-28578551

Fax: +852-25595337

Supplementary Method

For data analysis using Mothur software: 1) we cut the number of sequences to 61327 (the least sequences in one of the 26 treatments) for every treatment and combined them in one fasta file; 2) the sequences containing barcoded primers were trimmed and the primers and barcodes were cut out then; 3) 77260 unique tags were obtained from the nearly 1.6 million tags; 4) The unique tags were aligned using SILVA bacterial reference sequences as template (http://www.mothur.org/wiki/Silva_reference_files); 5) distances between the tags were calculated; 6) the tags were clustered (furthest clustering) at exact 0.04 cutoff which allows two bases distinction between two sequences with 52~75 bp, under which the number of total OTUs was 33187; 7) the tags were subsampled at the same depth according to the group containing the least tags (some tags were excluded during above operation); 8) a matrix describing the distributing of total 29533 OTUs in each treatment and a list of the names of tags for each OTU were obtained.

Notably, since we found it was hardly possible to detect chimera for V6 tags that are usually only 50~70 bp in length and theoretically few chimeras would be produced in short region PCR, no operations to exclude potential chimeras were performed. To evaluate the potential chimera in the final dataset, one hundred randomly selected tags (one representative of each of the 100 OTUs) were blasted against the NCBI database. Two potential chimeras were found due to their query coverage were less than 30% and the others were all covered more than 95% (i.e., less than 4 uncovered bases). The two OTUs are both singleton. It suggested there were few potential chimeras (around 80 chimeras within ~46000 reads of each treatment) and they had little impact on the final results, especially for the dominant groups.

Figure captions:

Figure S1. The fluorescent image of activated sludge from Stanley (A) and Shatin (B) wastewater treatment.

Figure S2. Electrophoresis of the extracted genomic DNA. For each marked kit name, the left lane is the as-is sample, and the right lane is the fixed sample. The results showed that DNA obtained from all the treatments were between 9 kb to 21 kb. DNA extracted by three kits, i.e. ZR-SM, QG-ST and EP-SM, were too low to be observable.

Figure S3. Rarefaction curves of the two fixed and as-is AS samples. A) The Stanley as-is sample; B) the fixed Stanley sample; C) the Shatin as-is sample; and D) the fixed Shatin sample.

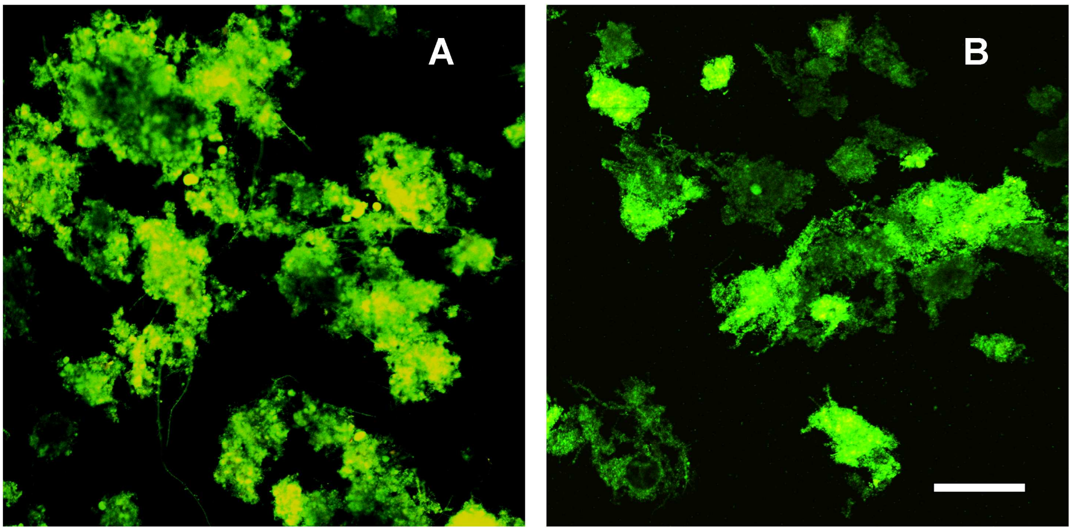

Figure S1. The SYBR green I stained activated sludge from Stanley (A) and Shatin (B) wastewater treatment.

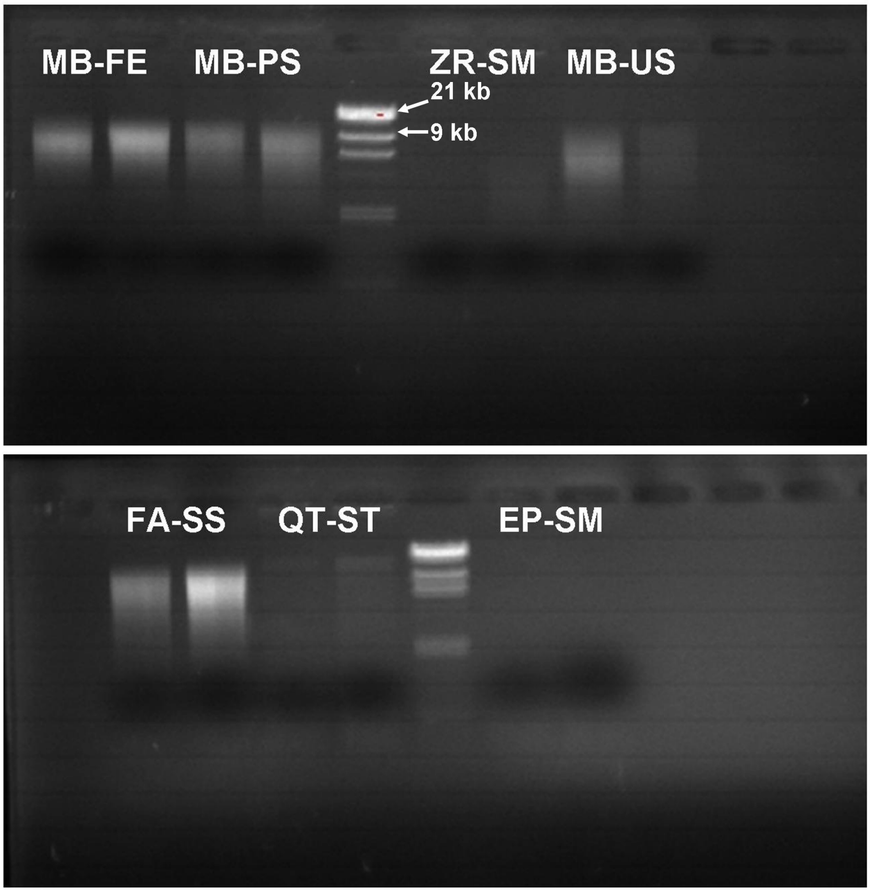

Figure S2. Electrophoresis of the extracted genomic DNA. For each marked kit name, the left lane is the as-is sample, and the right lane is the fixed sample. The results showed that DNA obtained from all the treatments were between 9 kb to 21 kb. DNA extracted by three kits, i.e. ZR-SM, QG-ST and EP-SM, were too low to be observable.

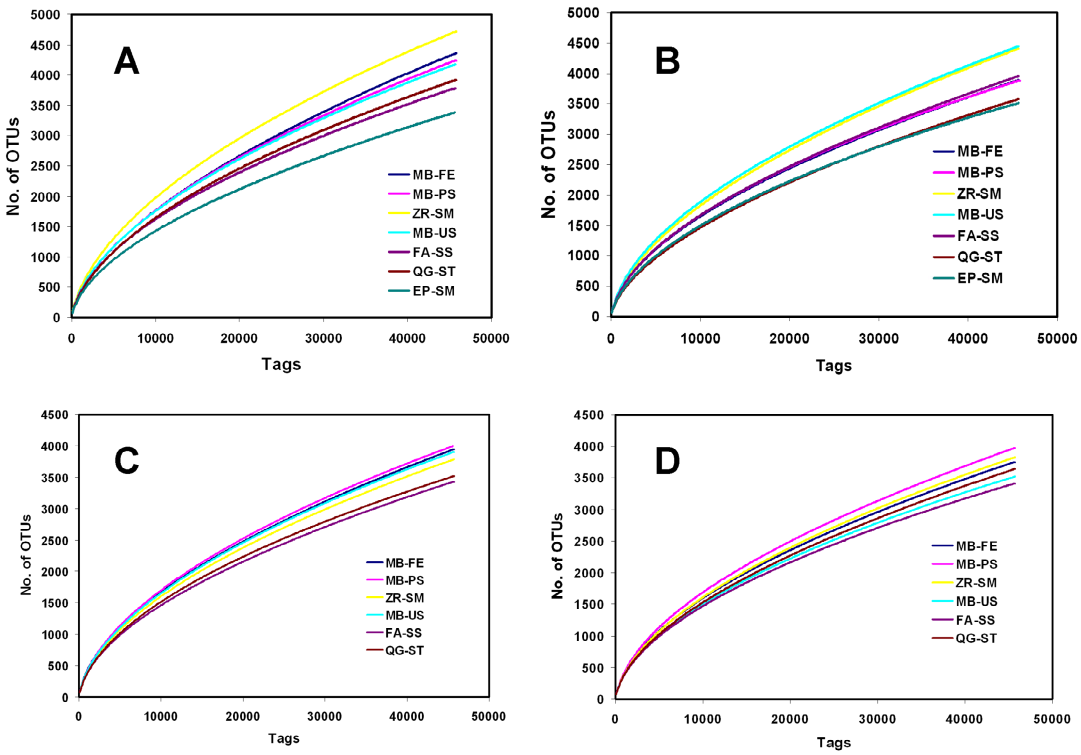

Figure S3. Rarefaction curves of the two fixed and as-is AS samples. A) The Stanley as-is sample; B) the fixed Stanley sample; C) the Shatin as-is sample; and D) the fixed Shatin sample.
